# Glucocorticoids Mediate Short-Term High-Fat Diet Induction of Neuroinflammatory Priming, the NLRP3 Inflammasome, and the Danger Signal HMGB1

**DOI:** 10.1523/ENEURO.0113-16.2016

**Published:** 2016-08-30

**Authors:** Julia L. Sobesky, Heather M. D’Angelo, Michael D. Weber, Nathan D. Anderson, Matthew G. Frank, Linda R. Watkins, Steven F. Maier, Ruth M. Barrientos

**Affiliations:** Department of Psychology and Neuroscience, and the Center for Neuroscience, University of Colorado Boulder, Boulder, Colorado 80309-0345

**Keywords:** : danger-associated molecular patterns, hippocampus, neuroimmunology, neuroinflammation, neuroinflammatory priming, short-term high-fat diet

## Abstract

The impact of the foods we eat on metabolism and cardiac physiology has been studied for decades, yet less is known about the effects of foods on the CNS, or the behavioral manifestations that may result from these effects. Previous studies have shown that long-term consumption of high-fat foods leading to diet-induced obesity sensitizes the inflammatory response of the brain to subsequent challenging stimuli, causing deficits in the formation of long-term memories. The new findings reported here demonstrate that short-term consumption of a high-fat diet (HFD) produces the same outcomes, thus allowing the examination of mechanisms involved in this process long before obesity and associated comorbidities occur. Rats fed an HFD for 3 d exhibited increases in corticosterone, the inflammasome-associated protein NLRP3 (nod-like receptor protein 3), and the endogenous danger signal HMGB1 (high-mobility group box 1) in the hippocampus. A low-dose (10 μg/kg) lipopolysaccharide (LPS) immune challenge potentiated the neuroinflammatory response in the hippocampus of rats fed the HFD, and caused a deficit in the formation of long-term memory, effects not observed in rats fed regular chow. The blockade of corticosterone action with the glucocorticoid receptor antagonist mifepristone prevented the NLRP3 and HMGB1 increases in unchallenged animals, normalized the proinflammatory response to LPS, and prevented the memory impairment. These data suggest that short-term HFD consumption increases vulnerability to memory disruptions caused by an immune challenge by upregulating important neuroinflammatory priming and danger signals in the hippocampus, and that these effects are mediated by increases in hippocampal corticosterone.

## Significance Statement

American adults consume diets that are higher in saturated fats and/or refined sugars [i.e., a high-fat diet (HFD)] than ever before, and these diets have been associated with significant cognitive deficits. This study aims to examine the mechanisms that underlie this relationship. Here, we demonstrate that short-term HFD consumption elevates the neuroinflammatory priming signals corticosterone, NLRP3 (nod-like receptor protein 3), and HMGB1 (high-mobility group box 1) in the hippocampus. When followed by an immune challenge, HFD produced a potentiated proinflammatory response and memory deficit. Inhibiting corticosterone signaling during HFD consumption prevented the priming, the potentiated neuroinflammation, and the memory impairment. Together, these data suggest that the glucocorticoid receptor is an important target for attenuating the neuroinflammatory effects associated with HFDs.

## Introduction

American adults consume diets higher in saturated fats and/or refined sugars [i.e., a high-fat diet (HFD)] than ever before (Beilharz et al., 2015; Guyenet and Carlson, 2015), and these diets have been associated with cognitive deficits in humans and rodents (Eskelinen et al., 2008; Ross et al., 2009; Kanoski et al., 2010; Solfrizzi et al., 2010; Heyward et al., 2012).

It has been proposed that HFD-associated neuroinflammation may be a mechanism that underlies cognitive deficits in overweight and obese individuals (Beilharz et al., 2014, 2015; Marissal-Arvy et al., 2014; Miller and Spencer, 2014; Sobesky et al., 2014; Spagnuolo et al., 2015; Stranahan, 2015), and this is a plausible proposal given the large body of literature implicating neuroinflammation as a cause of memory declines (Rachal Pugh et al., 2001; Yirmiya and Goshen, 2011; Barrientos et al., 2015b). However, because there are numerous comorbidities associated with obesity, studies directed at understanding the underlying mechanisms of obesity-induced memory declines can be easily confounded (Guh et al., 2009).

A growing literature has demonstrated that HFD consumption can cause memory impairments in humans and rodents in as little as 3-5 d, long before frank signs of obesity appear (Kanoski and Davidson, 2010; Holloway et al., 2011; Beilharz et al., 2014, 2016). These findings support the notion that the macronutrient profile of foods may be as important for cognitive health as is obesity status or total energy intake. Moreover, they provide a context in which inflammatory mechanisms could be examined without confounding by other comorbid conditions.

It is well known that HFD consumption induces inflammation in peripheral tissues through active secretion of adipokines by adipocytes (Coppack, 2001; Xu et al., 2002; Cano et al., 2009). Importantly, peripheral inflammation is capable of signaling the brain, via various routes of communication (Konsman et al., 2002), leading to *de novo* production of cytokines in the brain that can then alter behavior (Layé et al., 1994). Saturated free fatty acids have been shown to directly pass into the hypothalamus, where they activate toll-like receptor 4 (TLR4), producing a proinflammatory response there and causing behavioral modifications (Milanski et al., 2009; Maric et al., 2014). However, for reasons that remain unclear, free fatty acids do not pass directly into the hippocampus (Milanski et al., 2009).

While HFD consumption alone has been shown to induce proinflammatory gene expression in various brain regions, including hippocampus (Hansen et al., 1998; Thaler et al., 2012; Beilharz et al., 2014, 2016), it should be noted that these studies specifically included a substantial sugar component in their high-fat diet regimen, which may be a critical factor. A larger body of literature (from studies using saturated HFDs that do not have high sugar contents, such as the present one), suggests that hippocampal cells are primed by HFD consumption, and that a secondary challenge must occur before neuroinflammatory cytokines are detected or memory impairments are observed (Boitard et al., 2014; Cai et al., 2014; Knight et al., 2014; Sobesky et al., 2014). These studies have shown that HFD consumption alone does not produce elevated cytokine expression in the brain, but does elevate microglial markers of activation. Moreover, short-term HFD consumption sensitizes the hypothalamus and hippocampus to over-respond to an immune challenge, such as to lipopolysaccharide (LPS), and, in turn, produces functional impairments mediated by those brain regions. However, little is known about the mechanisms that mediate this short-term HFD-induced priming effect, and thus is the focus of the present study.

Here, we explored the novel idea that short-term consumption of HFD would induce an elevation in hippocampal corticosterone (CORT), which would in turn prime the hippocampus to amplify its inflammatory response to a mild inflammatory challenge, finally resulting in impairments in memory consolidation.

Despite its classic role as an immunosuppressant, there is a growing literature demonstrating that CORT can prime hippocampal microglia (Frank et al., 2010a, 2014; Barrientos et al., 2015a) and potentiate the neuroinflammatory response to a subsequent inflammatory challenge (Frank et al., 2010a; Munhoz et al., 2010; Hains et al., 2011; Loram et al., 2011).

Here, we demonstrate that short-term HFD consumption produces CORT elevations in the hippocampus, increases the expression of neuroinflammatory priming signals, potentiates the proinflammatory response to LPS, and causes a deficit in forming long-term memory. To test that this HFD-induced CORT increase is a critical mechanism in this cascade, we administered the GR antagonist mifepristone at the time of HFD intake. If this treatment would prevent an HFD-plus-LPS-induced potentiated neuroinflammatory response and memory impairment, this would provide new insight into the mechanisms underlying the impact of HFD consumption on cognitive declines.

## Materials and Methods

### Animals

Male Wistar rats (Harlan Laboratories) were used. All animals were ∼3 months of age and weighed between 275 and 375 g at the time of arrival. Following arrival, animals were allowed to acclimate to the facility for at least 7 d prior to diet modifications. Subjects were pair housed in standard large cages [52 × 30 × 21 cm (length [L] × width [W] × height [H])] with food and water administered *ad libitum*. The colony room was maintained at 22°C on a 12 h light/dark cycle (lights on at 7:00 A.M.). All experiments were conducted in accordance with protocols approved by the University of Colorado Animal Care and Use Committee.

### Diet

Animals were randomly assigned to one of three diets. One group (the regular group) continued consuming regular chow [catalog #TD.8640, Envigo; energy density, 3.0 kcal/g; 29% calories from protein; 54% from carbohydrates (no sweetener added), and 17% from fat (0.9% saturated, 1.2% monounsaturated, 2.7% polyunsaturated)]. A second group (the control group) was fed a control diet matched to the regular diet in macronutrients [catalog #TD.2029x, Envigo; energy density of 3.1 kcal/g; 24% calories from protein, 60% from carbohydrates (no sweetener added), and 16% from fat]. This group was included to control for the effects that may be present due to switching to a novel diet for 3 d. A third group (the HFD group) was fed an adjusted-calorie high-fat diet [catalog #TD.06414, Envigo; energy density of 5.1 kcal/g; 18.4% calories from protein, 21.3% from carbohydrates (90 g/kg sucrose, 160 g/kg maltodextrin), and 60.3% from fat (37% saturated, 47% monounsaturated, 16% polyunsaturated)]. Since there were no differences between the regular and control groups with regard to the data examined in experiment 1 (CORT levels, cytokine levels, or expression of the priming genes; see Results), for the remainder of the study only the regular chow group was compared with the HFD group.

### Glucose, insulin, and leptin measurement

Glucose (2 h fasting) was measured in whole blood using a commercial glucometer (Contour, Bayer). Plasma insulin was measured using a commercial ELISA kit for rat insulin from Abnova with a detection range of 0–140 μl/U/mL, and a sensitivity of <5 μl/U/mL. Plasma leptin was measured using a commercial ELISA kit for rat leptin from Millipore with an intra-assay variability of <3%. The detection limit of the assay is 0.08 ng/ml.

### LPS injections

LPS (*Escherichia coli*, serotype 0111:B4; Sigma-Aldrich), a potent TLR4 agonist, was used to induce an inflammatory response. LPS was administered intraperitoneally at a dose of 10 μg/kg, or saline served as the vehicle control. The dose of LPS was selected as it has shown to induce by itself only a subthreshold proinflammatory response in the hippocampus (Johnson et al., 2002).

### Mifepristone injections

To block the signaling activity of CORT, the glucocorticoid receptor (GR) antagonist mifespristone (Sigma-Aldrich) was dissolved in 100% propylene glycol and administered subcutaneously at a dose of 50 mg/kg/ml. Propylene glycol (100%) was used as the vehicle control.

### Tissue collection

Rats were injected intraperitoneally with a lethal dose of sodium pentobarbital until unresponsive and transcardially perfused with ice-cold 0.9% saline for 3 min. Following saline perfusion, brains were extracted from skull and placed on a clean glass dish inverted on ice, wherein hippocampus was dissected, placed into prelabeled 1.5 ml Eppendorf tubes, and flash frozen in liquid nitrogen. All samples were stored at −80° C until further processed.

### PCR

#### RNA isolation from whole tissue samples

RNA was isolated from whole tissue using a standard method of phenol-chloroform extraction (Chomczynski and Sacchi, 1987). Briefly, tissue samples were rapidly homogenized in 1 ml of TRIzol reagent (Invitrogen). Samples were homogenized using a Tissue Tearor homogenizer. After incubation at room temperature for 5 min, chloroform was added to the supernatant, vortexed for 2 min, and centrifuged (at 4°C, 12,000 × *g*, for 15 min) to achieve phase separation of nucleic acid. Isopropyl alcohol (0.5 volume of the TRIzol volume) was added to precipitate nucleic acid. Samples were briefly vortexed and incubated at room temperature for 10 min followed by centrifugation (at 4°C, 12,000 × *g*) for 10 min. Nucleic acid precipitate was washed in 75% ethanol (1 ml) and centrifuged (at 4°C, 7500 × *g*, for 5 min). The ethanol was gently poured out, the RNA pellet was allowed to dry, and resuspension was performed with 40 μl of nuclease-free water (Ambian).

#### cDNA synthesis of whole tissue-derived RNA

Total RNA was reverse transcribed into cDNA using the SuperScript II First Strand Synthesis System for RT-PCR (Invitrogen). A standard amount of sample was added to nucleic acid-free water to equate 11 μl. This RNA was incubated for 5 min at 65°C in a total reaction volume of 13 μl containing random hexamer primers (5 ng/μl) and deoxynucleotides (dNTPs; 1 mm). Samples were chilled on ice for at least 1 min. A cDNA synthesis buffer (6 μl) was added to the reaction and incubated at 20°C for 2 min. Reverse transcriptase (1 μl; 200 units of SuperScript II) was added to the reaction and incubated at 25°C for 10 min followed by 42°C for 50 min. Reaction was terminated by heating to 70°C for 15 min.

#### Primer specifications

cDNA sequences were obtained from the GenBank at the National Center for Biotechnology Information [NCBI (www.ncbi.nlm.nih.gov)]. Primer sequences were designed using the Eurofins MWG Operon Oligo Analysis and Plotting Tool (http://www.operon.com/technical/toolkit.aspx) and tested for sequence specificity using the Basic Local Alignment Search Tool at NCBI (Altschul et al., 1997). Primers were obtained from Invitrogen, and primer specificity was verified by melt curve analysis. Gene function and primer sequences of the genes of interest are presented in Table 1.

**Table 1:** PCR primer description and sequences

Gene	Primer sequence: 5' → 3'	Function
β-Actin	F: TTCCTTCCTGGGTATGGAAT R: GAGGAGCAATGATCTTGATC	Cytoskeletal protein (housekeeping gene)
IL-1β	F: CCTTGTGCAAGTGTCTGAAG R: GGGCTTGGAAGCAATCCTTA	Proinflammatory cytokine
IL-6	F: AGAAAAGAGTTGTGCAATGGCA R: GGCAAATTTCCTGGTTATATCC	Proinflammatory cytokine
IκBα	F: CACCAACTACAACGGCCACA R: GCTCCTGAGCGTTGACATCA	Marker for transcription factor NF-κB activity
CD11b	F: CTGGGAGATGTGAATGGAG R: ACTGATGCTGGCTACTGATG	Macrophage/microglial antigen marker
HMGB1	F: GAGGTGGAAGACCATGTCTG R: AAGAAGAAGGCCGAAGGAGG	Endogenous danger signal
NLRP3	F: AGAAGCTGGGGTTGGTGAATT R: GTTGTCTAACTCCAGCATCTG	Rate limiting protein in NLRP3 inflammasome formation
CX3CR1	F: TCAGGACCTCACCATGCCTA R: CGAACGTGAAGACAAGGGAG	Microglia-selective chemokine receptor
TLR4	F: TCCCTGCATAGAGGTACTTC R: CACACCTGGATAAATCCAGC	Pattern recognition receptor

CD, cluster of differentiation; F, forward; R, reverse.

#### Quantitative real-time PCR

PCR amplification of cDNA was performed using the Quantitect SYBR Green PCR Kit (Qiagen). cDNA (1 μl) was added to a reaction master mix (25 μl) containing 2.5 mm MgCl_2_, HotStar Taq DNA polymerase, SYBR Green I, dNTPs, fluorescein (10 nm), and gene-specific primers (500 nm each of forward and reverse primer). For each experimental sample, triplicate reactions were conducted in 96-well plates (Bio-Rad). PCR cycling conditions consisted of a hot-start activation of HotStart Taq DNA polymerase (at 94°C, for 15 min) and 40 cycles of denaturation (at 95°C, for 15 s), annealing (at 55–58°C, for 30 s), and extension (at 72°C, for 30 s). A melt curve analysis was conducted to assess the uniformity of product formation, primer dimmer formation, and the amplification of nonspecific products. The PCR product was denatured (at 95°C, for 1 min) and annealed (at 55°C, for 1 min) prior to melt curve analysis, which consisted of incrementally increasing reaction temperature (0.5°C/10 s) from 55°C to 95°C.

#### Real-time detection and relative quantification of PCR product

The formation of PCR product was monitored in real time using the MyiQ Single-Color Real-Time PCR Detection System (Bio-Rad). The Fluorescence of SYBR Green I was captured at 72°C. The threshold for the detection of PCR product above background was set at 10× the SD of the mean background fluorescence for all reactions. Background fluorescence was determined from cycle 1 to five cycles prior to the exponential amplification of product, and subtracted from the raw fluorescence of each reaction/cycle. The threshold for the detection of PCR product fell within the exponential phase of amplification for each reaction. The threshold cycle (C_T_; number of cycles to reach the threshold of detection) was determined for each reaction.

#### Relative quantitation of gene expression

Relative gene expression was determined using the 2^-ΔΔC^_T_ method (Livak and Schmittgen, 2001). The mean C_T_ of triplicate measures was computed for each sample. Sample mean C_T_ of the internal control (β-actin) was subtracted from the sample mean C_T_ of the respective gene of interest (ΔC_T_). The sample with the absolute highest mean ΔC_T_ was selected as a calibrator, and the mean ΔC_T_ of each experimental sample (ΔΔC_T_) was subtracted from this value. 2^ΔΔC^_T_ yielded a fold change in gene expression of the gene of interest normalized to the internal control gene expression and relative to the calibrator sample. Relative gene expression for each sample was calculated, and data are presented as the percentage of regular diet values.

### Hippocampus processing for ELISA and Western blot

In preparation for assays, tissue samples were sonicated in 0.3 ml sonication buffer (Invitrogen). Tissues were then mechanically homogenized using an ultrasonic cell disrupter (Thermo Fisher Scientific). Sonication consisted of 20 s of cell disruption at 52% amplitude. Sonicated samples were centrifuged (at 4°C, 10,000 × *g*, for 10 min), and supernatants were removed and stored at 4°C until ELISA or Western blots were performed. Bradford protein assays determined the total protein concentrations of sonicated tissue.

### Interleukin-1β and CORT

Levels of interleukin (IL)-1β protein and CORT were determined using commercially available rat-specific ELISA for IL-1β (R&D Systems) and corticosterone kits (Enzo Life Sciences). The assays were performed according to the manufacturer instructions. IL-1β was determined and normalized to total protein.

### Western blot

Samples were heated to 75°C for 10 min then loaded into a standard polyacrylamide Bis-Tris gel (Invitrogen). SDS-PAGE was performed in MOPS running buffer (Invitrogen) at 175 V for 75 min. Protein was transferred onto a nitrocellulose membrane using the iBlot dry transfer system (Invitrogen). The membrane was blocked with Odyssey blocking buffer (LI-COR) for 1 h and incubated with a primary antibody in blocking buffer overnight at 4°C. The following day, the membrane was washed in 1× PBS containing Tween 20 (0.1%) and then incubated in blocking buffer containing either goat anti-rabbit or goat anti-mouse (LI-COR) IRDye 800CW secondary antibody at a concentration of 1:10,000 for 1 h at room temperature. Primary antibodies included the following: mouse anti-rat high-mobility group box 1 (HMGB1) monoclonal antibody (1:4000; Abcam); rabbit anti-rat nod-like receptor protein 3 (NLRP3) monoclonal antibody (1:1000; Millipore); and mouse anti-rat β-actin (1:200,000; Sigma-Aldrich). Protein expression was quantified using an Odyssey Infrared Imager (LI-COR) and normalized to the housekeeping protein value for that sample, and data are presented as the percentage of the within-blot regular diet control samples.

### Context pre-exposure fear conditioning

A context pre-exposure fear-conditioning paradigm was used to measure memory performance, as this paradigm has been shown to be highly sensitive to disruptions to the hippocampus (Rudy et al., 2002; Matus-Amat et al., 2004). Contextual fear conditioning depends on the following two processes: the construction of a conjunctive representation of the conditioning context; and the association of that representation with shock. Acquiring a conjunctive representation depends on an intact hippocampus. Because in this paradigm the two processes are engaged independently (on separate days), it allows more accurate detection of impairments selective to the hippocampus (Matus-Amat et al., 2004). The conditioning context was one of two identical Igloo ice chests [54 × 30 × 27 cm (L × W × H)] with white interiors. A fan and an activated 24 V DC light bulb were mounted on the ceiling of each chest. The conditioning chambers [26 × 21 × 24 cm (L × W × H)], placed inside each chest, were made of clear plastic and had window screen tops. Chambers were cleaned with water before each animal was conditioned or tested.

Rats were taken two at a time from their home cage and transported to the conditioning context in a black ice bucket with the lid on so that the rats could not see where they were being taken. Rats were placed in the context and allowed to freely explore, and then they were transported back to their home cage, where they remained for ∼40 s before the next exposure. This procedure was repeated six times. Rats remained in the conditioning context for 5 min on the first exposure and for 40 s on the five subsequent exposures. The rats were transported in the black bucket each time that they were returned to their home cage, but with the lid off, so that they could discern whether they were headed to the context or to their home cage. The purpose of these multiple exposures was to establish the features of the black bucket as retrieval cues that could activate the representation of the context. Immediately after the last exposure, rats received an injection of saline or LPS (as described earlier). Seventy-two hours later, each animal was taken from its home cage and transported to the conditioning context in the black bucket again. There, they immediately received one 2 s footshock (1.5 mA delivered through a removable floor of stainless steel rods 1.5 mm in diameter, spaced 1.2 cm center to center). Each rod was wired to a shock generator and scrambler (Coulbourn Instruments). They were then quickly taken out of the chamber and transported back to their home cage. The time that the rats spent in the conditioning context never exceeded 5 s. A day later, the rats were placed back in the conditioning context, and memory was assessed by observing freezing behavior over a 6 min period. Two hours later, rats were placed in a novel, neutral, control context and observed for 6 min to detect any generalized fear or anxiety. After placing the rat into the chamber, every 10 s each rat was judged as either freezing or active at the instant the sample was taken. Freezing, the dominant defensive fear response of the rat, is a complete suppression of behavior that is accompanied by immobility, shallow breathing, and a variety of other autonomic changes, including an increase in heart rate and pilo-erection (Kim and Fanselow, 1992). Freezing in these experiments was defined as the absence of all visible movement, except for respiration. Scoring was performed by observers blind to experimental treatment, and inter-rater reliability exceeded 97% for all experiments.

### Data analysis

All data are presented as the mean ± SEM. Statistical analyses were computed using GraphPad Prism version 6 and StatView version 5. All experiments had six to eight rats per group. One-way ANOVAs were used for the analyses in experiment 1. Two-way ANOVAs were run for the analyses in experiment 2. Three-way ANOVAs were run for the analyses in experiments 3 and 4. In the case of a significant interaction, *post hoc* tests were run. The threshold for significance was set at α = 0.05.

## Results

### Short-term HFD consumption increases body mass

**Figure 1.**
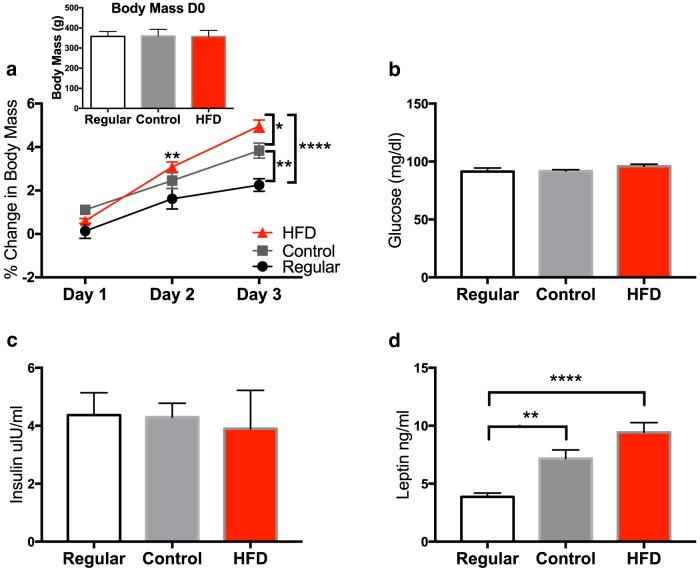
Body mass and metabolic changes. ***a***, Daily percentage change in body mass over the 3 d in which rats had free access to the regular chow diet, control diet, and the HFD. Inset, Body weights on day 0 of rats assigned to the three groups. ***b–d***, Levels of whole-blood glucose (***b***), plasma insulin (***c***), and plasma leptin (***d***) in rats fed a regular chow diet, a control diet, and an HFD. Data are reported as the mean ± SEM. *p < 0.05; **p < 0.01; ***p < 0.001; ****p < 0.0001.

To determine whether short-term consumption of an HFD would significantly increase body mass, animals from the three diet groups were weighed on the day of diet switch (day 0) and every day for 3 d. Body weight averages for all three groups at the start of the experiment were not different (*p* = 0.98; Fig. 1, inset; Table 2). A repeated-measures ANOVA was run to analyze the percentage of weight gained among the three diets across the 3 d. A significant interaction (diet × time) effect (*F*_(4,42)_ = 8.49, *p* < 0.0001; Fig. 1*a*) led to *post hoc* analyses revealing a significant increase in the HFD group compared with the regular group on day 2 (*p* < 0.01). On day 3, the HFD group gained more than the regular group (*p* < 0.0001) and the control group (*p* < 0.05). In addition, the control group gained significantly more than the regular group (*p* < 0.01).

**Table 2:** Statistics

Figure	Data Structure	Type of test	*p* Values
1 (inset)	Normal distribution	One-way ANOVA	0.98
1a	Normal distribution	Two-way repeated-measures ANOVA (time × diet) Tukey’s *post hoc* test Day 1 Regular vs control Regular vs HFD Control vs HFD Day 2 Regular vs control Regular vs HFD Control vs HFD Day 3 Regular vs control Regular vs HFD Control vs HFD	<0.0001 95% CI −2.022 to 0.077 −1.518 to 0.581 −0.546 to 1.553 −1.887 to 0.211 −2.513 to −0.414 −1.675 to 0.424 −2.632 to −0.533 −3.762 to −1.663
1b	Normal distribution	One-way ANOVA	0.31
1c	Normal distribution	One-way ANOVA	0.93
1d	Normal distribution	One-way ANOVA Tukey’s *post hoc* test Regular vs control Regular vs HFD Control vs HFD	<0.0001 95% CI −5.787 to −0.827 −8.047 to −3.086 −4.740 to −0.221
2	Normal distribution	One-way ANOVA Fisher’s LSD *post hoc* test Regular vs control Regular vs HFD Control vs HFD	0.028 95% CI −0.378 to 0.180 −0.658 to −0.100 −0.559 to −0.001
3a	Normal distribution	One-way ANOVA	0.194
3b	Normal distribution	One-way ANOVA	0.131
3c	Normal distribution	One-way ANOVA Fisher’s LSD *post hoc* test Regular vs control Regular vs HFD Control vs HFD	0.026 95% CI −29.17 to 9.432 −46.26 to −7.658 −35.74 to 1.556
3d	Normal distribution	One-way ANOVA Fisher’s LSD *post hoc* test Regular vs control Regular vs HFD Control vs HFD	0.049 95% CI −16.12 to 16.28 −34.89 to −1.426 −34.44 to −2.038
3e	Normal distribution	One-way ANOVA Fisher’s LSD *post hoc* test Regular vs control Regular vs HFD Control vs HFD	0.026 95% CI −55.03 to 47.63 −104.2 to −1.550 −98.77 to 0.406
3f	Normal distribution	One-way ANOVA Fisher’s LSD *post hoc* test Regular vs control Regular vs HFD Control vs HFD	0.017 95% CI −21.42 to 15.11 −45.09 to −7.351 −41.33 to −4.796
4a	Normal distribution	Two-way ANOVA Diet Challenge Diet × challenge Scheffé’s *post hoc* test Regular:saline vs regular:LPS Regular:saline vs HFD:saline Regular:LPS vs HFD:LPS HFD:saline vs HFD:LPS	0.002 <0.0001 0.040 0.008 0.071 0.010 0.0003
4b	Normal distribution	Two-way ANOVA diet challenge diet × challenge Scheffé’s *post hoc* test Regular:saline vs regular:LPS Regular:saline vs HFD:saline Regular:LPS vs HFD:LPS HFD:saline vs HFD:LPS	0.040 <0.0001 0.031 0.0014 0.778 0.042 0.0007
4c	Normal distribution	Two-way ANOVA diet challenge diet × challenge Scheffé’s *post hoc* test Regular:saline vs regular:LPS Regular:saline vs HFD:saline Regular:LPS vs HFD:LPS HFD:saline vs HFD:LPS	0.003 <0.0001 0.015 0.0003 0.091 0.013 0.0008
5a	Normal distribution	Three-way ANOVA Diet Treatment Challenge Diet × treatment Diet × challenge Treatment × challenge Diet × treatment × challenge Scheffé’s *post hoc* test Vehicle:saline:regular vs vehicle:saline:HFD Vehicle:LPS:regular vs vehicle:LPS:HFD Mife:saline:regular vs Mife:saline:HFD Mife:LPS:regular vs Mife:LPS:HFD Regular:saline:vehicle vs regular:saline:Mife Regular:LPS:vehicle vs regular:LPS:Mife HFD:saline:vehicle vs HFD:saline:Mife HFD:LPS:vehicle vs HFD:LPS:Mife Regular:vehicle:saline vs regular:vehicle:LPS Regular:Mife:saline vs regular:Mife:LPS HFD:vehicle:saline vs HFD:vehicle:LPS HFD:Mife:saline vs HFD:Mife:LPS	0.0002 0.0053 <0.0001 0.105 0.038 <0.0001 0.033 0.083 0.0005 0.235 0.531 0.100 0.152 0.126 0.003 <0.0001 0.346 <0.0001 0.323
5b	Normal distribution	Three-way ANOVA Diet Treatment Challenge Diet × treatment Diet × challenge Treatment × challenge Diet × treatment × challenge Scheffé’s *post hoc* test Vehicle:saline:regular vs vehicle:saline:HFD Vehicle:LPS:regular vs vehicle:LPS:HFD Mife:saline:regular vs Mife:saline:HFD Mife:LPS:regular vs Mife:LPS:HFD Regular:saline:vehicle vs regular:saline:Mife Regular:LPS:vehicle vs regular:LPS:Mife HFD:saline:vehicle vs HFD:saline:Mife HFD:LPS:vehicle vs HFD:LPS:Mife Regular:vehicle:saline vs regular:vehicle:LPS Regular:Mife:saline vs regular:Mife:LPS HFD:vehicle:saline vs HFD:vehicle:LPS HFD:Mife:saline vs HFD:Mife:LPS	0.572 0.543 <0.0001 0.002 0.211 0.171 0.003 0.173 0.011 0.364 0.133 0.088 0.115 0.500 0.014 <0.0001 0.003 <0.0001 0.004
5c	Normal distribution	Three-way ANOVA Diet Treatment Challenge Diet × treatment Diet × challenge Treatment × challenge Diet × treatment × challenge Scheffé’s *post hoc* test Vehicle:saline:regular vs vehicle:saline:HFD Vehicle:LPS:regular vs vehicle:LPS:HFD Mife:saline:regular vs Mife:saline:HFD Mife:LPS:regular vs Mife:LPS:HFD Regular:saline:vehicle vs regular:saline:Mife Regular:LPS:vehicle vs regular:LPS:Mife HFD:saline:vehicle vs HFD:saline:Mife HFD:LPS:vehicle vs HFD:LPS:Mife Regular:vehicle:saline vs regular:vehicle:LPS Regular:Mife:saline vs regular:Mife:LPS HFD:vehicle:saline vs HFD:vehicle:LPS HFD:Mife:saline vs HFD:Mife:LPS	0.015 0.426 <0.0001 0.0004 0.005 0.237 0.0014 0.946 0.001 0.348 0.589 0.026 0.119 0.078 0.011 0.0005 0.032 <0.0001 0.031
5d	Normal distribution	Three-way ANOVA Diet Treatment Challenge Diet × treatment Diet × challenge Treatment × challenge Diet × treatment × challenge Scheffé’s *post hoc* test Vehicle:saline:regular vs vehicle:saline:HFD Vehicle:LPS:regular vs vehicle:LPS:HFD Mife:saline:regular vs Mife:saline:HFD Mife:LPS:regular vs Mife:LPS:HFD Regular:saline:vehicle vs regular:saline:Mife Regular:LPS:vehicle vs regular:LPS:Mife HFD:saline:vehicle vs HFD:saline:Mife HFD:LPS:vehicle vs HFD:LPS:Mife Regular:vehicle:saline vs regular:vehicle:LPS Regular:Mife:saline vs regular:Mife:LPS HFD:vehicle:saline vs HFD:vehicle:LPS HFD:Mife:saline vs HFD:Mife:LPS	0.145 0.043 <0.0001 0.003 0.361 0.173 0.289 0.027 0.063 0.049 0.435 0.075 0.751 0.017 0.027 0.001 0.006 0.005 0.002
5e	Normal distribution	Three-way ANOVA Diet Treatment Challenge Diet × treatment Diet × challenge Treatment × challenge Diet × treatment × challenge Scheffé’s *post hoc* test Vehicle:saline:regular vs vehicle:saline:HFD Vehicle:LPS:regular vs vehicle:LPS:HFD Mife:saline:regular vs Mife:saline:HFD Mife:LPS:regular vs Mife:LPS:HFD Regular:saline:vehicle vs regular:saline:Mife Regular:LPS:vehicle vs regular:LPS:Mife HFD:saline:vehicle vs HFD:saline:Mife HFD:LPS:vehicle vs HFD:LPS:Mife Regular:vehicle:saline vs regular:vehicle:LPS Regular:Mife:saline vs regular:Mife:LPS HFD:vehicle:saline vs HFD:vehicle:LPS HFD:Mife:saline vs HFD:Mife:LPS	0.009 0.226 0.01 0.006 0.942 0.044 0.060 0.010 0.027 0.130 0.037 0.145 0.072 0.939 0.021 0.010 0.017 0.091 0.75
5f	Normal distribution	Three-way ANOVA Diet Treatment Challenge Diet × treatment Diet × challenge Treatment × challenge Diet × treatment × challenge Scheffé’s *post hoc* test Vehicle:saline:regular vs vehicle:saline:HFD Vehicle:LPS:regular vs vehicle:LPS:HFD Mife:saline:regular vs Mife:saline:HFD Mife:LPS:regular vs Mife:LPS:HFD Regular:saline:vehicle vs regular:saline:Mife Regular:LPS:vehicle vs regular:LPS:Mife HFD:saline:vehicle vs HFD:saline:Mife HFD:LPS:vehicle vs HFD:LPS:Mife Regular:vehicle:saline vs regular:vehicle:LPS Regular:Mife:saline vs regular:Mife:LPS HFD:vehicle:saline vs HFD:vehicle:LPS HFD:Mife:saline vs HFD:Mife:LPS	0.017 0.083 0.061 0.0007 0.269 0.111 0.459 <0.0001 0.136 0.760 0.149 0.074 0.375 0.049 0.0007 0.0001 0.876 0.338 0.535
6a	Normal distribution	Three-way ANOVA Diet Treatment Challenge Diet × treatment Diet × challenge Treatment × challenge Diet × treatment × challenge Scheffé’s *post hoc* test Vehicle:saline:regular vs vehicle:saline:HFD Vehicle:LPS:regular vs vehicle:LPS:HFD Mife:saline:regular vs Mife:saline:HFD Mife:LPS:regular vs Mife:LPS:HFD Regular:saline:vehicle vs regular:saline:Mife Regular:LPS:vehicle vs regular:LPS:Mife HFD:saline:vehicle vs HFD:saline:Mife HFD:LPS:vehicle vs HFD:LPS:Mife Regular:vehicle:saline vs regular:vehicle:LPS Regular:Mife:saline vs regular:Mife:LPS HFD:vehicle:saline vs HFD:vehicle:LPS HFD:Mife:saline vs HFD:Mife:LPS	0.908 0.377 0.197 0.177 0.126 0.342 0.035 0.234 0.012 0.772 0.440 0.735 0.456 0.631 0.014 0.476 0.688 0.002 0.904
6b	Normal distribution	Three-way ANOVA Diet Treatment Challenge Diet × treatment Diet × challenge Treatment × challenge Diet × treatment × challenge	0.154 0.722 0.297 0.157 0.518 0.740 0.051

Mife, Mifepristone.

### Short-term HFD consumption effects on metabolic measures

To determine whether short-term consumption of an HFD would significantly alter metabolic function, glucose, insulin, and leptin were measured in animals from the three diet groups. A one-way ANOVA was run for each measure. Levels of glucose (*F*_(2,15)_ = 1.29, *p* = 0.31; Fig. 1*b*) and insulin (*F*_(2,15)_ = 075, *p* = 0.93; Fig. 1*c*) were not different among the three groups. The leptin level (*F*_(2,15)_ = 17.19, *p* = 0.0001; Fig. 1*d*) was significantly elevated in both the control (*p* < 0.01) and HFD (*p* < 0.0001) groups compared with the regular chow group. The control and HFD groups did not differ from each other (*p* = 0.08).

### Experiment 1: short-term HFD consumption produces neuroinflammatory priming

**Figure 2.**
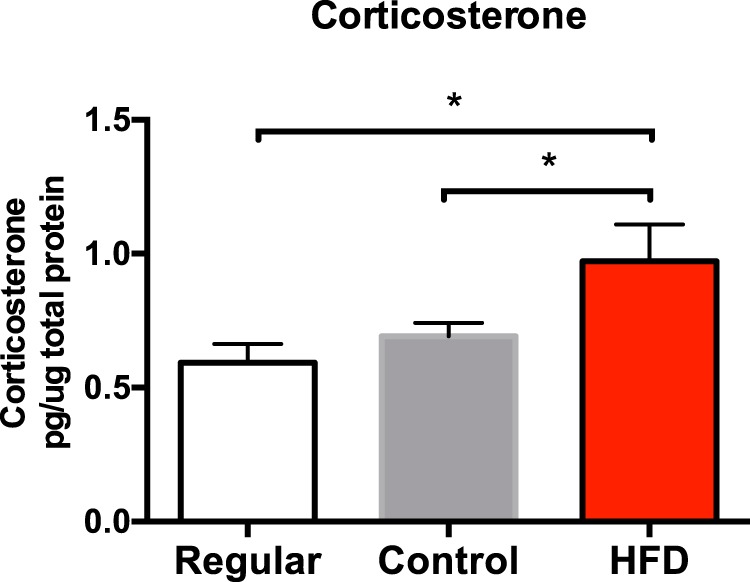
Hippocampal CORT levels among rats fed a regular chow diet, a control diet, or an HFD. Data are reported as the mean ± SEM. *p < 0.05.

**Figure 3.**
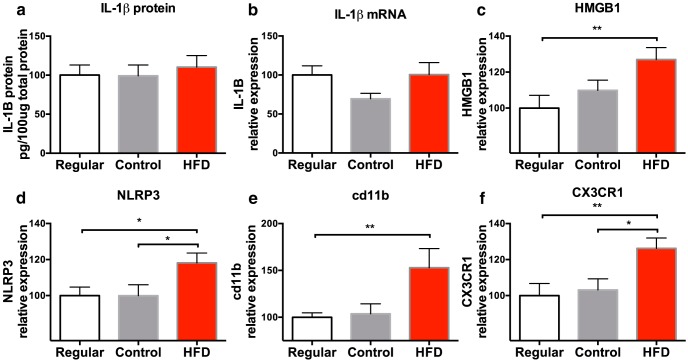
***a–f***, Hippocampal levels of IL-1β protein (***a***), and mRNA expression of IL-1β (***b***), HMGB1 (***c***), NLRP3 (***d***), cd11b (***e***), and CX3CR1 (***f***) of rats fed a regular chow diet, a control diet, or an HFD. Data are reported as the mean ± SEM. All mRNA levels are relative to regular chow diet values. *p < 0.05; **p < 0.01.

Following 3 d on their respective diets, rats were taken from their home cage and killed, and whole hippocampal tissue was collected and processed to measure CORT; the IL-1 inflammasome-associated protein NLRP3; the endogenous danger-associated molecular signal HMGB1; the pan-macrophage activation marker cd11bl chemokine (fractalkine) receptor 1 (CX3CR1), which is selectively expressed on microglia; IL-1β; and the pattern recognition receptor TLR4. These analytes were chosen to serve as markers of an inflammatory phenotype. It should be noted that CORT was measured in the hippocampus rather than in the circulation because, although brain levels of CORT usually reflect the levels of CORT in the circulation, incongruencies have been reported (Barrientos et al., 2015a), and since the focus of this study is on the hippocampus, measuring CORT levels specifically in the hippocampus was ideal. Hippocampal CORT levels were significantly higher in rats that were fed the HFD compared with levels in both the regular (*p* < 0.01) and control (*p* < 0.05) diet groups (*F*_(2,18)_ = 4.39, *p* < 0.05; Fig. 2). CORT levels did not differ between the regular and control groups (*p* > 0.05). There were no significant differences in IL-1β protein (*F*_(2,23)_ = 0.42, *p* > 0.05; Fig. 3*a*) or gene expression (*F*_(2,21)_ = 2.24, *p* > 0.05; Fig. 3*b*), or in TLR4 gene expression (*F*_(2,15)_ = 0.26, *p* = 0.77; not shown) in the hippocampus among the groups. Short-term consumption of an HFD caused significant increases in gene expression of the endogenous danger signal HMGB1 (*F*_(2,20)_ = 4.41, *p* < 0.05; Fig. 3*c*), NLRP3 (*F*_(2,19)_ = 3.54, *p* < 0.05; Fig. 3*d*), the macrophage activation marker CD11b (*F*_(2,20)_ = 4.42, *p* < 0.05; Fig. 3*e*), and the fractalkine receptor CX3CR1, which is selectively expressed on microglia (*F*_(2,19)_ = 5.12, *p* < 0.05; Fig. 3*f*).

### Experiment 2: short-term HFD consumption potentiates inflammatory response to LPS

**Figure 4.**
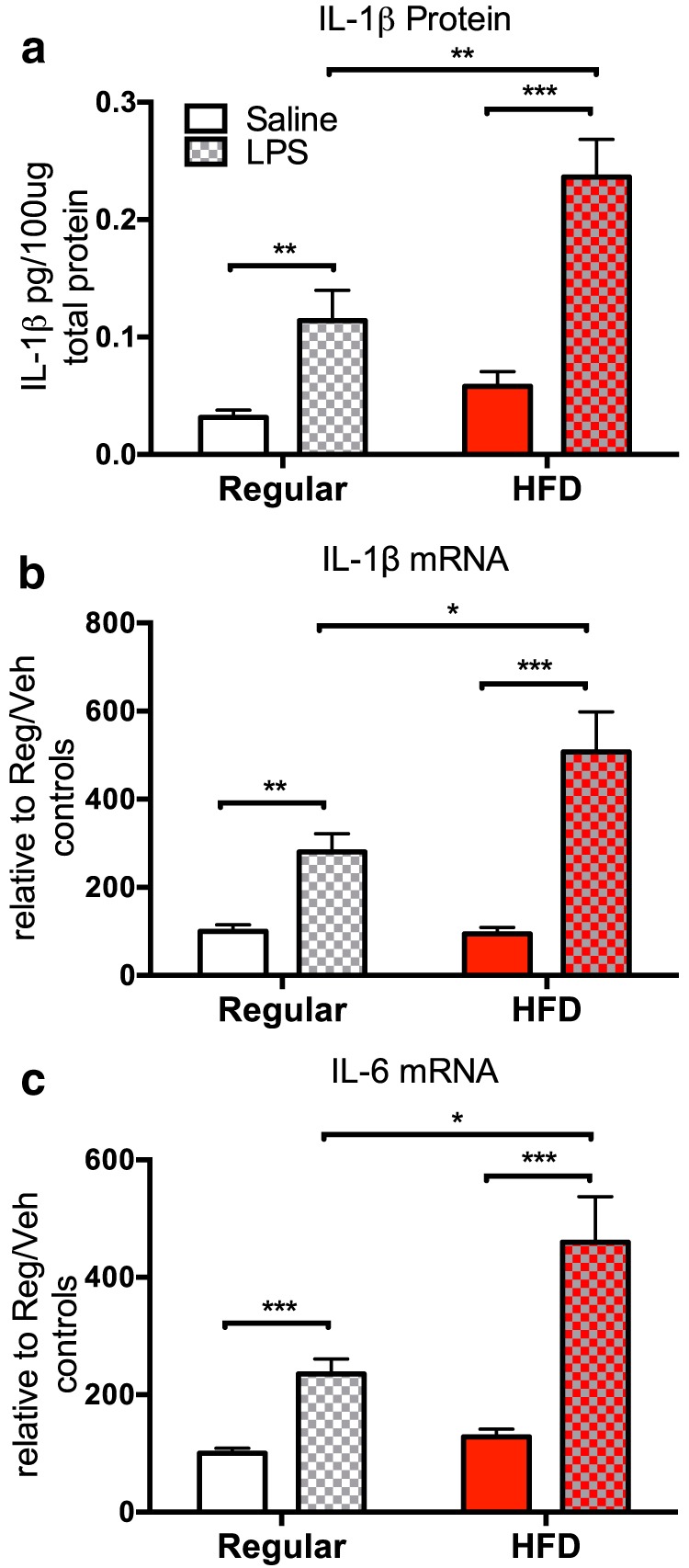
***a–c***, Hippocampal expression levels of proinflammatory cytokines IL-1β protein (***a***), IL-1β mRNA (***b***), and IL-6 mRNA (***c***) of rats fed a regular chow diet or an HFD 2 h following an injection of peripheral saline or LPS. Data are reported as the mean ± SEM. *p < 0.05; **p < 0.01; ***p < 0.001.

To evaluate whether short-term HFD consumption would amplify the hippocampal proinflammatory response to a subsequent immune challenge, LPS or saline was administered to rats on the third day of consuming their respective diets, and were killed 2 h later. Hippocampi were collected and processed to measure the expression of the proinflammatory cytokines IL-1β and IL-6. LPS by itself produced increases in hippocampal IL-1b protein, and IL-1 and IL-6 mRNA. Importantly, the consumption of HFD exaggerated these increases to LPS. IL-1β protein levels were potentiated in the hippocampus of the HFD plus LPS group (*F*_(1,27)_ = 4.64, *p* < 0.05; Fig. 4*a*) compared with levels exhibited by the regular plus LPS group (*p* < 0.01). Similarly, IL-1β (*F*_(1,24)_ = 5.24, *p* < 0.05; Fig. 4*b*) and IL-6 (*F*_(1,23)_ = 6.90, *p* < 0.05; Fig. 4*c*) gene expression were potentiated in the HFD plus LPS group compared with levels exhibited by the regular plus LPS group (*p* < 0.05).

### Experiment 3: mifepristone attenuates HFD plus LPS-induced potentiated neuroinflammation

**Figure 5.**
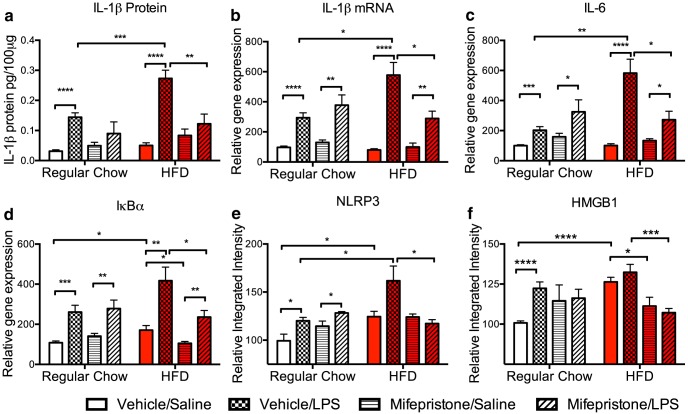
***a–f***, Hippocampal expression levels of IL-1β protein (***a***), IL-1β mRNA (***b***), IL-6 mRNA (***c***), IκBα mRNA (***d***), NLRP3 protein (***e***), and HMGB1 protein (***f***) 2 h following a saline or LPS injection among rats fed a regular chow diet or an HFD and treated with vehicle or mifepristone. Data are reported as the mean ± SEM relative to regular chow diet values. *p < 0.05; **p < 0.01; ***p < 0.001; ****p < 0.0001.

Glucocorticoids mediate the neuroinflammatory priming that is produced by stress (Frank et al., 2012) and aging (Barrientos et al., 2015a). Thus, given the observation in experiment 1 that 3 d of HFD consumption increases hippocampal levels of CORT and induces a primed neuroinflammatory phenotype, as well as an exaggerated response to subsequent LPS consumption (experiment 2), we explored the hypothesis that increased signaling by CORT during HFD consumption mediates the impact of the HFD consumption on neuroinflammation. To test this hypothesis, we administered peripheral injections of the blood–brain-permeable GR antagonist mifepristone (Schreiber et al., 1983) or vehicle to rats fed a regular chow diet or an HFD. Although the goal was to block GR signaling in hippocampus, peripheral administration was deemed satisfactory, as peripheral mifepristone administration at this dose has previously been demonstrated to be effective in preventing CORT-mediated neuroinflammatory priming within hippocampus (Frank et al., 2012). Injections were given 24 and 48 h after the initiation of diet (half-life of mifepristone, ∼18 h). On the third day of HFD consumption, rats were given an injection of either LPS or a saline vehicle and were killed 2 h later. Hippocampi were collected and processed to measure IL-1β, IL-6, NLRP3, HMGB1, and IκBα [nuclear factor κ light chain enhancer of activated B-cell inhibitor α (a measure of nuclear factor-κB [NF-κB] activation)]. NF-κB is a transcription factor that regulates the transcription of many inflammatory cytokines. Mifepristone treatment effectively reduced the potentiated expression of all of these proinflammatory markers induced by HFD and LPS. Three-way ANOVAs were used to analyze these data. A significant diet (regular vs HFD) × treatment (vehicle vs mifepristone) × challenge (saline vs LPS) interaction effect on IL-1β protein was found (*F*_(1,50)_ = 4.800, *p* < 0.05; Fig. 5*a*). *Post hoc* tests revealed that within the regular chow group, vehicle plus LPS-treated rats exhibited higher levels of IL-1β protein than vehicle plus saline-treated controls (*p* < 0.0001). Similarly, within the HFD group, vehicle plus LPS-treated rats exhibited higher levels of IL-1β protein than did vehicle plus saline-treated controls (*p* < 0.0001). Within the vehicle plus LPS groups, HFD-fed rats exhibited higher levels of IL-1β protein than regular chow-fed controls (*p* < 0.001), replicating the findings from experiment 2. In support of our hypothesis, we found that within the HFD plus LPS groups, mifepristone treatment produced a significant decrease in IL-1β protein compared with vehicle-treated rats (*p* < 0.01). Similar results were observed with all of the molecules we examined. Significant interaction effects were observed for IL-1β mRNA (*F*_(1,50)_ = 9.891, *p* < 0.01; Fig 5*b*), IL-6 mRNA (*F*_(1,50)_ = 11.512, *p* < 0.01; Fig 5*c*), IκBα mRNA expression (*F*_(1,51)_ = 9.794, *p* < 0.01; Fig 5*d*), NLRP3 protein expression (*F*_(1,44)_ = 8.187, *p* < 0.01; Fig 5*e*), and HMGB1 protein expression (*F*_(1,46)_ = 5.23, *p* < 0.05; Fig 5*f*). All *post hoc* significant differences between groups are depicted in the figures. Most notable of these is the significant decrease in expression in the mifepristone-treated rats within the HFD plus LPS groups compared with the vehicle-treated rats (IL-1β, *p* < 0.05; IL-6, *p* < 0.05; IκBα, *p* < 0.05; NLRP3, *p* < 0.05; HMGB1, *p* < 0.001).

### Experiment 4: mifepristone attenuates HFD plus LPS-induced contextual memory impairments

**Figure 6.**
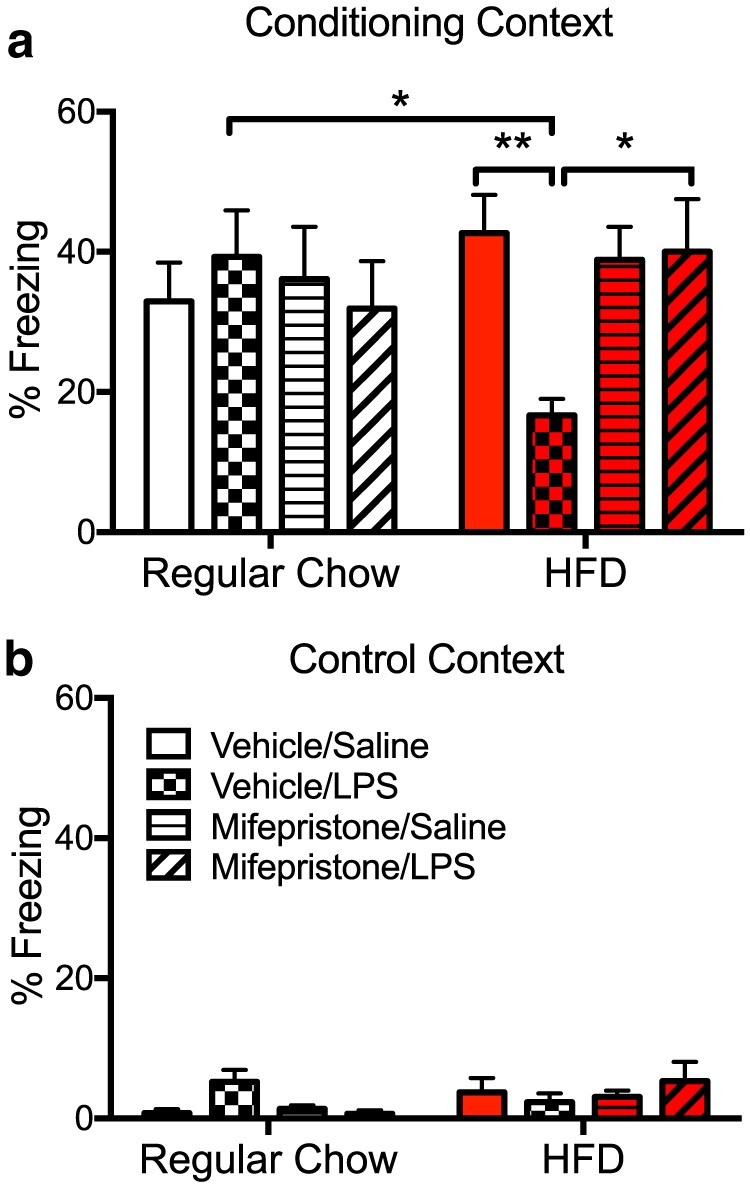
***a***, ***b***, Percentage of freezing to the conditioning context (***a***) or the control (neutral) context (***b***) among rats fed a regular chow diet or an HFD, treated with vehicle or mifepristone, and challenged with saline or LPS. Data are reported as the mean ± SEM. *p < 0.05; **p < 0.01.

In this experiment, two questions were addressed. First, we determined whether short-term HFD intake followed by an immune challenge would impair contextual memory. We also determined the role of CORT signaling in any such impaired memory function. Previous findings have shown that increases in proinflammatory cytokines in the hippocampus produce impairments in hippocampal-dependent memory function (Rachal Pugh et al., 2001; Barrientos et al., 2002). Thus, given that the interaction of short-term HFD intake and LPS challenge produces potentiated levels of hippocampal IL-1, we tested the impact of these challenges on contextual memory function. Moreover, to test the hypothesis that elevations in hippocampal CORT level contribute to this effect, we administered mifepristone, as in the previous experiment, to determine whether this treatment would attenuate any impairments caused by diet and LPS administration. On the third day on their respective diets, rats were pre-exposed to the conditioning context, during which a contextual representation is normally formed (see Procedural details). Immediately following this procedure, rats received either LPS or saline. Three days later, rats were brought back to the conditioning chamber and given a 2 s footshock and taken immediately back to their home cage. The next day, rats were placed back into the conditioning chamber and tested for memory of context fear. LPS did not interfere with the development of context fear memory in rats fed the regular chow diet, nor did mifepristone have any effect. However, 3 d of eating an HFD led LPS potently impairing the development of context fear memory, and this impairment was prevented by mifepristone. A three-way ANOVA was used to analyze these data. A significant diet (regular vs HFD) × treatment (vehicle vs mifepristone) × challenge (saline vs LPS) interaction effect on freezing behavior was found (*F*_(1,42)_ = 4.742, *p* < 0.05; Fig. 6*b*). In support of the first hypothesis, *post hoc* tests revealed that within the vehicle plus LPS groups, HFD-fed rats froze significantly less than did regular chow-fed controls (*p* < 0.05). Within the HFD group, vehicle plus LPS-treated rats froze significantly less than did vehicle plus saline-treated controls (*p* < 0.01). In support of the second hypothesis, within the HFD plus LPS groups, mifepristone treatment produced a significant increase in freezing behavior compared with vehicle-treated rats (*p* < 0.05). Data examining freezing behavior in the control context produced no main effect of diet, treatment, or challenge (*p* > 0.05; Fig 6*a*). There were also no significant interaction effects (*p* > 0.05). These data demonstrate that rats did not show any generalized fear leading to greater freezing.

## Discussion

Although rats were fed the HFD for only 3 d, they gained a significant amount of weight over that gained in rats fed the regular and control diets. Interestingly, rats that were fed the control diet also exhibited a significant increase compared with rats fed the regular diet, suggesting that at least a portion of the increased body mass exhibited by the HFD group could be attributed to the novelty of the diet, as the consumption of a novel diet has been shown to induce weight gain after an initial (1 d) period of neophobia (Eckel and Ossenkopp, 1993; Modlinska et al., 2015). Taking this factor into account, the HFD group gained 1% body weight above that of the control diet group. Note that this is only 3-4 g. Nonetheless, these findings are consistent with what others have found after short-term consumption of a high-fat diet (Hansen et al., 1998; Beilharz et al., 2014, 2016; Cai et al., 2014). Glucose and insulin were not at all different among the three diet groups. Leptin levels, however, were elevated in both the control and HFD groups compared with the regular chow group. Interestingly, similar HFD-induced leptin increases were reported in rats that had been fed the same HFD used here for 5 months (Sobesky et al., 2014), suggesting that long-term HFD is not necessary to induce leptin increases. The fact that the control diet produced an increase in leptin, but did not produce increases in corticosterone, HMGB1, NLRP3, cd11b, or CX3CR1 suggests that increased leptin levels alone are not responsible for the primed inflammatory response in the hippocampus. It is not clear why the control diet, which is matched to the regular diet in macronutrients, would produce an increase in leptin, though a possibility is that this increase was triggered by the rapid and short-term novel diet-induced weight increase.

Despite the fact that both control-fed and HFD-fed groups demonstrated a body mass increase compared with the regular chow-fed group, only the HFD-fed group exhibited an elevation in hippocampal CORT levels. These data are consistent with other findings showing that short-term HFD consumption increases plasma CORT levels (Tannenbaum et al., 1997). Rats fed the control diet showed no such CORT elevation, eliminating the possibility that a novel diet alone produced this elevation in CORT levels. Classically, the immune-modulatory impacts of CORT have been viewed as primarily anti-inflammatory (De Bosscher and Haegeman, 2009). However, this notion is being updated as prior stress sensitizes proinflammatory responses to subsequent LPS (Johnson et al., 2002; Munhoz et al., 2006; Hains et al., 2011), and the proinflammatory effects of stress have been linked to CORT signaling (Munhoz et al., 2010; Frank et al., 2012). It appears that the timing of rises CORT in levels is important for determining the resulting anti-inflammatory or proinflammatory function, as CORT elevations prior to a secondary challenge increase the response to LPS, but CORT signaling after the inflammatory challenge dampens the response (Frank et al., 2010a).

Short-term HFD consumption alone did not produce increases in IL-1β protein or mRNA, confirming what others have found (Beilharz et al., 2014; Boitard et al., 2014; Cai et al., 2014; Sobesky et al., 2014). Perhaps if a substantial sugar component would have been added to the HFD, an elevated inflammatory response would have occurred, as others have reported (Hansen et al., 1998; Thaler et al., 2012; Beilharz et al., 2014, 2016). Short-term HFD consumption did, however, increase expression of the alarmin HMGB1 and the NLRP3 protein. These data are consistent with previous findings showing that levels of these molecules are elevated in response to increases in CORT levels (Busillo et al., 2011; Frank et al., 2014; Weber et al., 2015). HMGB1 is an endogenous danger signal, or danger-associated molecular pattern (DAMP), that can stimulate inflammation through interactions with a number of pattern recognition receptors, such as TLR2 and TLR4 (Yanai et al., 2012), to activate the proinflammatory transcription factor NF-κB (Su et al., 2011). NLRP3 is a structural component of one type of inflammasome, which regulates the cleavage and release of IL-1β through the activation of caspase-1 (Khare et al., 2010; Schroder and Tschopp, 2010). In particular, the NLRP3 inflammasome has been implicated in the mediation of inflammatory priming, as the activation of this inflammasome requires both a priming step and an activation step (Latz, 2010). It is important to note that HMGB1 has been demonstrated to increase NLRP3 mRNA and protein in hippocampus as well as microglia (Frank et al., 2016). Further, HMGB1 primed the neuroinflammatory and microglial proinflammatory response to a subsequent immune challenge (Frank et al., 2016). In light of these findings, a distinct possibility is that HMGB1 primed the NLRP3 inflammasome as a consequence of HFD treatment, but this possibility awaits testing.

It should be noted that, although these changes were not observed in microglia per se, we did observe increased hippocampal expression of the macrophage antigen cd11b, and the microglial-selective chemokine receptor CX3CR1, suggesting that HFD may indeed activate microglia, as others have found (Cai et al., 2014; Knight et al., 2014). However, future experiments will be aimed at determining the specific role of microglia in these HFD-induced changes. Together, these data suggest that although short-term consumption of HFD did not produce frank neuroinflammation in the hippocampus, it did produce increased expression of molecular signals that are known to prime the hippocampus to over-respond to a subsequent immune challenge. It is interesting to note that similar findings were reported following long-term HFD consumption (Sobesky et al., 2014), strongly suggesting that long-term HFD consumption is not necessary to produce a primed neuroinflammatory microenvironment.

Though short-term HFD alone was insufficient to induce an inflammatory response in the hippocampus, short-term HFD amplified hippocampal IL-1β and IL-6 increases to peripheral LPS. These data confirm that the inflammatory response in the hippocampus was indeed primed by HFD and are consistent with what others have found (Boitard et al., 2014; Cai et al., 2014; Sobesky et al., 2014). These findings provide a good basis to expect that short-term HFD consumption followed by a subsequent immune challenge would produce an impairment of hippocampal-dependent memory, as there is now a substantial literature confirming the role of proinflammatory cytokines in impairing hippocampal memory function (Rachal Pugh et al., 2001; Yirmiya and Goshen, 2011; Barrientos et al., 2015b). Indeed, the present data demonstrated that short-term HFD consumption alone did not impair memory, but when combined with a mild LPS immune challenge, HFD consumption robustly impaired contextual memory consolidation, a memory function specifically mediated by the hippocampus (Barrientos et al., 2002; Rudy et al., 2002; Matus-Amat et al., 2004).

In experiment 3, the GR antagonist mifepristone was administered at the time of diet consumption to evaluate the role of CORT signaling in the neuroinflammatory priming caused by short-term consumption of an HFD. The data strongly suggest that CORT is a key mediator of these effects. HFD potentiated the expression of IL-1 and IL-6 in LPS-injected rats, replicating the findings from experiment 2. Mifepristone treatment effectively normalized this proinflammatory response to LPS, leading to levels that resembled those of the regular chow-fed group. The data in this experiment also demonstrated that short-term HFD consumption produces a greater expression of NLRP3, HMGB1, and IκBα in the absence of LPS, replicating the findings from experiment 1 showing that HFD alone upregulates neuroinflammatory priming signals in the hippocampus. Mifepristone treatment, in unchallenged HFD-fed rats, significantly reduced the expression of HMGB1 and IκBα, but not of NLRP3. These data suggest that CORT directly induces HMGB1. Moreover, in LPS-challenged rats, mifepristone significantly reduced the expression of HMGB1 and IκBα, as well as of NLRP3. Of note, these findings are the first to demonstrate glucocorticoid modulation of levels of DAMPs either in the periphery or the CNS. Of course, it should be noted that MAP kinases (e.g., p38) and NF-κB are signal transduction pathways downstream of innate immune response receptors (e.g., pattern recognition receptors), which bind HMGB1 (Kawai and Akira, 2010), and therefore may play a role in mediating neuroinflammatory priming. Thus, these may be important targets to block these effects.

A key question arising from the present findings concerns how CORT induces HMGB1 in HFD-treated animals. One possibility is that elevated levels of CORT in brain may damage neural cells, leading to the passive release of HMGB1, which then signals microglia to generate a proinflammatory response. The basis for this speculation derives from work by Sapolsky (1999) demonstrating that glucocorticoids exacerbate the neurotoxic effects of excitotoxic agents, thereby “endangering” neurons.

Given the data from experiment 3 showing a robust inhibitory effect of mifepristone on neuroinflammatory priming signals as well as on the LPS-induced proinflammatory response, we expected and were not surprised to find that mifepristone treatment prevented the HFD plus LPS-induced memory impairments. These data are consistent with findings showing the following: (1) that the inhibition of proinflammatory signals in the hippocampus at the time of memory consolidation prevents memory impairments (Frank et al., 2010b; Barrientos et al., 2012); (2) protracted corticosterone release in HFD-fed adolescent rats alters amygdala-dependent cognitive function and neuronal plasticity (Boitard et al., 2015); and (3) mifepristone effectively reduces immune-activated proinflammatory responses, specifically from hippocampal microglia, and prevents *E. coli*-induced memory impairments in aged rats (Barrientos et al., 2015a).

The data presented here strongly implicate a role for CORT as a mediator of HFD-induced neuroinflammatory priming, and potentiated inflammatory responses to LPS that lead to contextual memory impairments. This is concluded because the inhibition of CORT signaling with mifepristone prevented all of these effects. Together, these data suggest that the glucocorticoid receptor may be an important target for attenuating the neuroinflammatory effects associated with HFDs.

## Synthesis

The decision was a result of the Reviewing Editor Leonidas Stefanis and the peer reviewers coming together and discussing their recommendations until a consensus was reached. A fact-based synthesis statement explaining their decision and outlining what is needed to prepare a revision is listed below. The following reviewers agreed to reveal their identity: Sophie Layé, Katia Karalis

Both reviewers agree that the study is very well executed and that the data are clear and provide some innovation. Both indicate that further work is needed to characterize the effects of the different diet regimens, and that additional molecular components should be investigated. Overall, further work is needed in order to strengthen the manuscript.

In particular, further measurements of various biochemical indices, such as p38, Asc1, and TLR4, will provide further insights. Western immunoblotting for NLRP3 is needed. The choice of the diet regimen and, importantly, the effects of the diet on the metabolic profile, including glucose, insulin, leptin, and lipids should be clarified and discussed within the context of the findings. Statements regarding the knowledge of the relationship between food consumption and behavior should be moderated. Further discussion of the findings in relation to the literature is warranted, as suggested by the reviewers.
